# Immunomodulatory Role and Therapeutic Potential of Non-Coding RNAs Mediated by Dendritic Cells in Autoimmune and Immune Tolerance-Related Diseases

**DOI:** 10.3389/fimmu.2021.678918

**Published:** 2021-07-29

**Authors:** Yifeng Liu, Xiaoze Wang, Fan Yang, Yanyi Zheng, Tinghong Ye, Li Yang

**Affiliations:** Department of Gastroenterology and Hepatology, Sichuan University-University of Oxford Huaxi Joint Centre for Gastrointestinal Cancer, West China Hospital, Sichuan University, Chengdu, China

**Keywords:** autoimmune disease, immune tolerance, dendritic cell, non-coding RNA, ce-RNAs

## Abstract

Dendritic cells (DCs) are professional antigen-presenting cells that act as a bridge between innate immunity and adaptive immunity. After activation, DCs differentiate into subtypes with different functions, at which point they upregulate co-stimulatory molecules and produce various cytokines and chemokines. Activated DCs also process antigens for presentation to T cells and regulate the differentiation and function of T cells to modulate the immune state of the body. Non-coding RNAs, RNA transcripts that are unable to encode proteins, not only participate in the pathological mechanisms of autoimmune-related diseases but also regulate the function of immune cells in these diseases. Accumulating evidence suggests that dysregulation of non-coding RNAs contributes to DC differentiation, functions, and so on, consequently producing effects in various autoimmune diseases. In this review, we summarize the main non-coding RNAs (miRNAs, lncRNAs, circRNAs) that regulate DCs in pathological mechanisms and have tremendous potential to give rise to novel therapeutic targets and strategies for multiple autoimmune diseases and immune tolerance-related diseases.

## Introduction

The first study of dendritic cells (DCs) was published in 1973, when Ralph Steinman and Zan Cohn discovered a small group of cells with unique stellate morphology by microscopic studies of glass-adhering mouse splenocytes (1). In the mononuclear phagocyte system (MPS), some MPS cells retain incompletely degraded antigen and present it to T cells, thus activating T cells (2). These so-called antigen-presenting cells (APCs) initiate a response by activating T cells, which subsequently stimulate antibody production from B cells, thus bridging innate immunity and adaptive immunity (3). DCs serve as a bridge between innate immunity and adaptive immunity, and the discovery of DCs is the result of efforts to understand the cellular initiating factors of the adaptive immune response (2).

Recent research shows that DCs can be classified into major subtypes based on origin and differentiation state. Human DCs are produced through a lymphoid-specific bone marrow haematopoiesis pathway. DC subset differentiation is affected by different specific transcription factors, among which the roles of IRF8 and IRF4 are particularly important (4–7). Under the regulation of these cellular transcription factors, DCs can differentiate into three main subgroups: plasmacytoid DCs (pDCs), type 1 myeloid/conventional DCs (cDC1s) and type 2 myeloid/conventional DCs (cDC2s) (8). In 2019, Brown et al. further classified cDC2s into cDC2A(T-bet^+^) and cDC2B(T-bet^-^) by assessing the expression of T-bet, and they are different from proinflammatory and anti-inflammatory phenotypes *in vivo (* 9). In addition, increasing evidence has shown that mature DCs can limit effector T cells and promote the differentiation of regulatory T (Treg) cells to promote the formation of immune tolerance in related diseases (10–12).

Researchers have found that genes encode not only functional products such as proteins but also a variety of unique RNAs (13). Despite a lack of protein-coding regions, *Caenorhabditis elegans* was found to carry some RNAs with conserved functions required for cell development (14). Owing to advances in sequencing technologies, researchers have found a large number of various non-coding RNAs. These non-coding RNAs can be divided into several subsets, including microRNAs (miRNAs), circular RNAs (circRNAs), long non-coding RNAs (lncRNAs), tRNA-derived small RNAs (tsRNAs), ribosomal RNAs (rRNAs), and PIWI-interacting RNAs (piRNAs) (14). Some highly conserved RNAs, including miRNAs (15), circRNAs, and lncRNAs, lacking conservation between species (16), account for approximately 60% of the transcriptional output of human cells (17, 18). It is clear that cellular processes and pathways can be regulated though non-coding RNAs in developmental and pathological settings.

Noncoding RNAs play various roles in the regulation of immune cell differentiation and function. Kuiper et al. observed that conditional depletion of Dicer in mouse CD11c+ DCs did not affect the presence of transient resident DCs in lymph nodes or spleen. However, the lack of miRNAs led to a selective loss of these cells in the epidermis, and those cells that did exist lacked the capacity to mature and present antigens (19). Wang et al. demonstrated that lnc-DCs, exclusively expressed in human conventional DCs (cDCs), decreased DC differentiation and reduced the antigen presentation ability of DCs by increasing the expression of STAT3 (20). Zhang et al. found that the expression of circular malat-1 (circ_malat-1) was attenuated by GDF15, leading to repression of the maturation of DCs (21).

Due to the unique role of DCs in immune diseases, researchers have paid more attention to the regulation of DCs by non-coding RNAs in recent years, considering this an important mechanism for further studying the relevant mechanisms and pathological processes in immune diseases. This review summarizes recent developments in non-coding RNA and DC research related to various autoimmune diseases and transplantation immunity, especially highlighting the immunomodulatory role of miRNAs, circRNAs, and lncRNAs in the processes of immune diseases mediated by DCs (**Table 1**).

**Table 1:** The targets and regulatory effect of noncoding RNAs on DCs in autoimmune and immune tolerance-related diseases.

Disease	Non-coding RNAs	Type of regulation	DCs (subsets or sources)	Predicted/identified targets	Function	Refs
SLE	miR574	↑	pDC	TLR7	Promote pDC maturation and secretion of IFN-α, TNF- and IL-6	(22)
	miR LET7b miR21					
	miR-361-5p,	↓	pDC	TLR7	Increase IFN-α secretion	(23)
	miR-128-3p miR-181a-2-3p					
	miR-155	↑	pDC	TLR7	MHC class II, CD40, CD86 expressions and IFN-α secretion increased	(24)
	miR-29b	↓	pDC	TLR9 Mcl-1, Bcl-2	Promote pDCs apoptosis	(25)
	miR-29c					
	miRNA-150	↓	cDC	TREM-1	inflammation decreased in SLE	(26)
	miR-142-3p	↑	cDC	ND	Increase secretion of related cytokines, inhibit Treg, and promote proliferation of CD4+T	(27)
RA	miR-34a	↑	DCs (CD1c^+^)	AXL	Promote DCs activation of T cells	(28)
	miR-363	↓	cDC (CD11C^+^av^+^)	ND	Increase Th17 cells differentiation	(29)
pSS	miR-29a	↓	pDC	ND	Increase pDCs survival	(30)
	mir-29c					
	miR-708	↓	cDC (CD1c^+^)	TLR3, TLR7/8	**Increase the secretion of IL-12 and TNF-α**	(31)
	miR-130a			MSK1		
IBD	miR-10a	↓	cDC (CD11c^+^)	IL-12/IL-23p40	Low inflammatory environment in the intestines	(32)
MS	miR-233	↓	cDC (CD11b^+^CD11c^+^)	ND	Inhibit activation of Th17 by decreasing levels of IL-1, IL-6, IL-23	(33)
SSc	miR-31	↑	cDC (CD11c^+^)	ND	Reduce the number of DC migrations to CNS	(34)
	miR-618	↑	pDC	IRF8	Reduce the development of pDCs in SSc	(35)
Autoimmune myocarditis	miR-223-3p	↑	Tol-DC	NLRP3	Inhibition of DCs maturation	(36)
GVHD	miR-155	↑	DCs (BMDC)	ND	Decrease the migration and inflammatory activation of DC	(37)
	miR-146a	↓	DCs (BMDC, MoDC)	JAK-STAT	Upgrade histopathological GVHD scores	(38)
	miR-29a	↑	DCs (BMDC, MoDC)	TLR7 (mouse)	promote DC maturation, migration and activation of T cell proliferation	(39)
				TLR8 (human)		
SLE	lnc-DC (ENST00000604411.1, ENST00000501122.2)	↑	DCs(MoDC)	ND	Positive correlation with SLEDAI Score	(40)
Autoimmune myocarditis	lncRNA NEAT1	↓	cDCs (CD80^+^, CD86^+^, MHC II^+^)	Sponge miR-3076-3p NLRP3	Increase DC induced Tregs and inhibited T cells proliferation	(41)
	lncRNA MALAT1	↑	Tol-DCs (DC-sign^+^)	mir155-5p	Promote the formation of Tol-DCs	(42)
SLE	circHLA-C	↑	DCs	miR-150	Promote pDCs maturation	(43)
Autoimmune myocarditis	circSnx5	↑	cDC (CD80^+^, CD86^+^, MHC II^+^)	miR-544	Reduce inflammation of EAM by regulating SOCS1, PU.1	(44)
	circ_Malat-1	↓	cDC (CD11c^+^CD80^+^, CD86^+^, MHC II^+^)	**GDF15**	Increase tolerogenic phenotype of DCs	(21)
				**NFκB**		

Increase the secretion of IL-12 and TNF-a; Increased IL-12 and TNF-A secretion in DCs. GDF15, Growth differentiation factor 15; NF-κB, nuclear factor kappa-B. ND, not done; ↑, upregulated; ↓, downregulated.

## Plasmacytoid Dendritic Cells (PDCs)

pDCs are a small subset of DCs that share a similar origin, and pDCs express a narrow range of pattern-recognition receptors (PRRs), including Toll-like receptor 7 (TLR7) and TLR9 (45). Under the stimulation of the above receptors and exogenous or endogenous nucleic acids, pDCs can secrete a large amount of type I IFN and other pro-inflammatory cytokines.

The numbers of pDCs in lymphoid tissues and related target organs, as well as the level of peripheral type I IFN, change in autoimmune diseases such as systemic lupus erythematosus (SLE), rheumatoid arthritis (RA) and psoriasis (46–48). In SLE, differentiation of Exfo B cells into AFCs requires activation of TRL signalling, which requires the involvement of pDCs (49). Some researchers, therefore, maintain that depletion or functional impairment of pDCs may serve as a viable and potentially specific treatment strategy for lupus (50). In addition to acting directly on autoimmune diseases, pDCs can also affect autoimmunity by regulating other immune cells. Nakamoto et al. demonstrated that bone marrow-derived pDCs induce IL-35 production through Treg cells during ConA-induced acute hepatitis, and the level of type I IFN released by pDCs was also increased. Consequently, the role of pDCs in autoimmune diseases cannot be ignored.

## Conventional Dendritic Cells (CDCs)

According to the dependence of transcription factors on development, different subtypes of cDC can be divided into cDC1 and cDC2 (51). In the MHC I environment, cDC1s present antigens to immature CD8+ T cells, while in the MHC II environment, cDC2s present more antigens to immature CD4+ T cells (52).

As cells that play a significant role in nonspecific and specific immunity, cDCs are also involved in a variety of autoimmune diseases. The number of cDCs in the peripheral blood of patients with autoimmune diseases (SLE or RA) is related to their localization in the target tissue (53–56). In RA patients, the number of cDCs was found to be increased in synovial fluid and decreased in peripheral blood (57). cDCs appear to express a unique chemokine receptor: CCL6, the CCL20 receptor. CCL20 leads to infiltration of a variety of inflammatory cells, including immature DCs and Th17 effector lymphocytes, and the production of inflammatory cytokines, including TNF-α, IL-1, and IL-17, in inflammatory synovial tissue, which induces recruitment of local cDCs (58, 59). We demonstrated that the role of abnormal autophagy in the immunogenic maturation of cDCs in autoimmune hepatitis should not be ignored, and inhibition of autophagy may be a novel therapeutic strategy for AIH (60).

## Tolerogenic Dendritic Cells (Tol-DCs)

DCs can promote the tolerance of autoreactive T cells and induce effector T cell differentiation in specific tissue environments, thus affecting autoimmunity, immune tolerance, or both (61). DCs in this state are called tolerogenic DCs (Tol-DCs). However, whether there is a specific sensitized cell origin in the body or whether the sensitized phenotype of DCs reflects their activation state is still unclear (62).

The role of Tol-DCs in autoimmunity is characterized by low expression of costimulatory molecules, production of immunomodulatory cytokines, and inhibition of the proliferation of T cells (63). In addition, the important interaction between Tregs and Tol-DCs in the maintenance of peripheral tolerance in mice and humans cannot be ignored (64). Tol-DCs can promote the differentiation of Treg cells through various mechanisms, such as the production of IL-10, IL-27, TGF and other cytokines and the expression of indoleamine 2,3-dioxygenase (IDO), thereby changing the levels of extracellular adenosine triphosphate (ATP) and adenosine (12, 65–68). Furthermore, treatment centred on tol-DCs administration is yielding promising results as an alternative to immune modulators (69). Tolerant dendritic cells inhibited T cell proliferation and delayed the occurrence of GVHD in mice through lactic acid synthesis (70).

## MicroRNAs Regulate Dendritic Cell-Mediated Autoimmune and Immune Tolerance-Related Diseases

**Figure 1:**
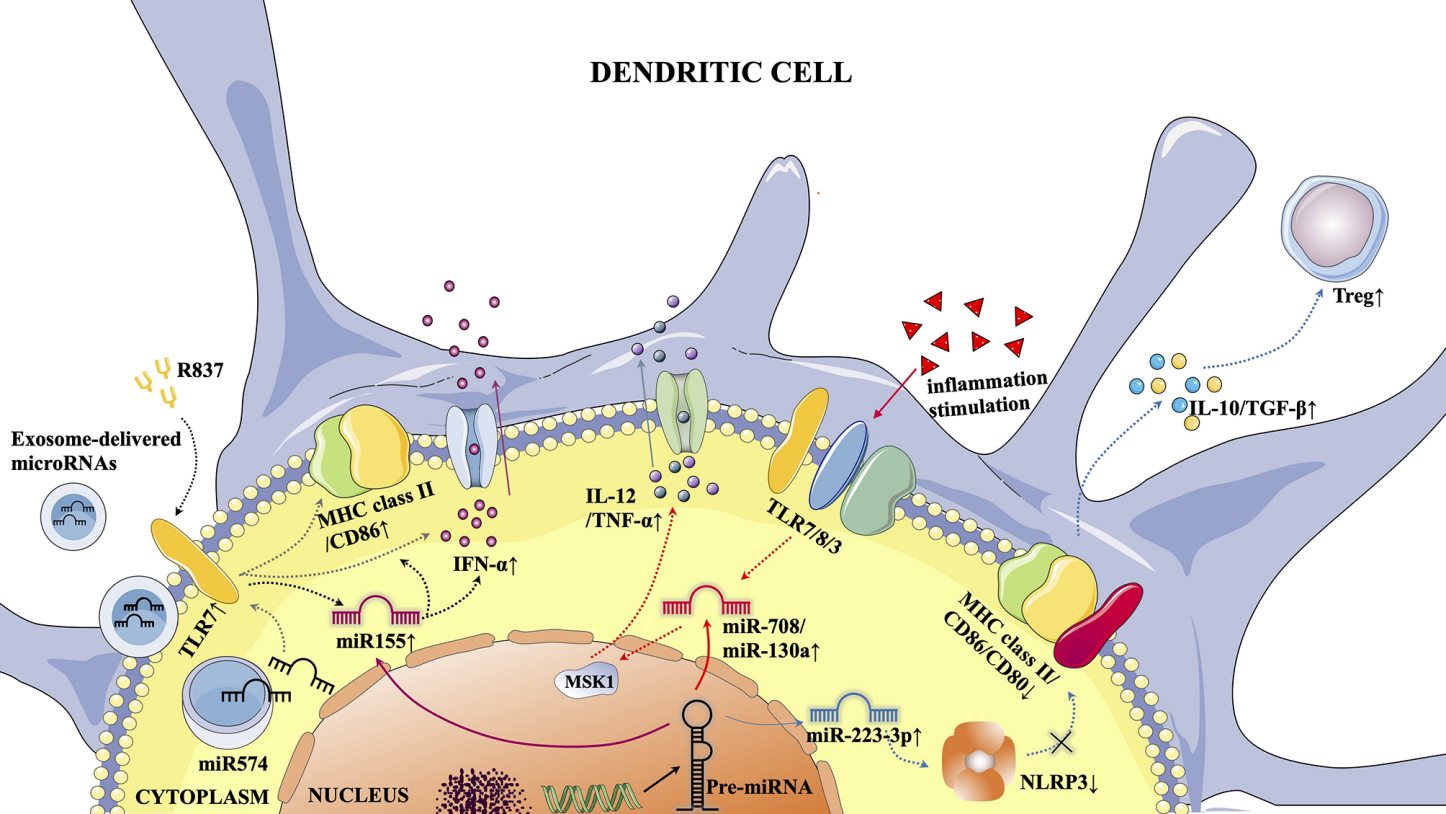
Typical microRNA-mediated pathways in DCs. 1) Activation of TLR7 by the TLR7 agonist R837 resulted in increased miR155 expression, which in turn promoted pDC maturation (elevated MHC class II/CD86 expression) and increased IFN-α secretion. 2) Under the stimulation of extraneous inflammatory factors, TLR7/8/3 was activated, which increased the expression of miR-708/miR-130a, leading to the inhibition of MSK1 and promoting the secretion of IL-12/TNF-α in cDCs. 3) The increased expression of miR-223-3p was followed by inhibition of NLRP3 inflammocytes, thereby promoting the DC tolerance phenotype (decreased EXPRESSION of MHC Class II/CD86/CD80), leading to increased secretion of IL-10/TGF-β and promoting Treg proliferation. 4) Activation of TLR7 by exosome-derived microRNAs through cell membranes can promote pDC maturation and increase IFN-α secretion.

Some previous studies have shown that miRNAs can act as regulatory molecules to affect the expression of target genes, thereby altering the immune state of the body (71). MiRNAs influence the pathogenesis of a variety of autoimmune and immune tolerance-related diseases by regulating DCs (**Figure 1**). In terms of treatment, pri-miRNAs may even become innovative drugs for the treatment of immune diseases (72).

### Systemic Lupus Erythematosus (SLE)

The cause of SLE is multifactorial, including the environment, random factors and genetic susceptibility (73). Large amounts of type I IFN and various cytokines produced by pDCs are typically found to be statistically related to the aetiopathogenesis of SLE (74). Salvi et al. purified exosomes from plasma collected from SLE patients and extracted miRNAs (idiopathic inflammatory myopathy (IIM) miRNAs: miR574, LET7b, and miR21) that could induce the production of type I IFNs in human pDCs from these exosomes. These miRNAs can act as survival factors for human pDCs, activate the maturation of pDCs, increase the expression of CD86 and decrease BDCA-2 levels as well as the production of IFN and pro-inflammatory cytokines (TNF-α, IL-6) and phosphorylated p65 (a subunit of NF-κB). Moreover, IIM miRNAs represent potential endogenous ligands of human TLR7, which is the specific endosomal single-stranded RNA (ssRNA) receptor expressed by pDCs (22). Hoogen et al. analysed 131 miRNAs in pDCs in SLE and related diseases (SLE + antiphospholipid syndrome and primary antiphospholipid syndrome) and found that 73 of them showed reduced expression. Of the 73 miRNAs, miR-361-5p, miR-128-3p and miR-181a-2-3p were expressed at lower levels in patients with a high IFN signature than in patients with a low IFN signature and healthy controls (23). By employing pDCs from murine models of lupus, Tam et al. discovered that the upregulation of miR-155 was the strongest, and the upregulation of miR-155 was significantly higher in active pDCs from the symptomatic group than in those from the control group. In agreement with this, TLR7-mediated miR-155 overexpression has been shown to lead to elevated CD40 expression (24). This finding is consistent with another study showing that MHC class II, CD40, and CD86 expression is decreased by miR-155 knockdown in Kupffer cells (75). pDCs activated by the TLR pathway are resistant to glucocorticoid-induced apoptosis, which makes glucocorticoids ineffective in the treatment of type I IFN-related autoimmune diseases. In another study, miR-29b and miR-29c promoted pDC apoptosis by directly targeting Mcl-1 and Bcl-2, which elevated the therapeutic effect of glucocorticoids in SLE (25). TLR and IFN receptors are innate immune receptors, and dysregulation of TLR and IFN signalling can lead to innate immune system disorders; these pathways have been shown to be important in lupus pathogenesis (76). As we have previously described, dysregulated miRNAs influence the progression of SLE by regulating pDCs activated by TLRs and/or IFN, as well as by inducing the secretion of inflammatory cytokines.

Not only pDCs but also active cDCs play important roles in the development of SLE. Triggering receptor expressed on myeloid cells-1 (TREM-1) might play a part in the pathogenesis of autoimmune disorders such as lupus through TLR-induced inflammatory responses (77). By selecting and analysing splenocytes from MRL/lpr mice, Gao et al. found that the expression of miR-150 could downregulate the levels of TREM-1, suggesting that TREM-1 may be a therapeutic target for the prevention of inflammatory cDC effects in SLE (26). In addition, miR-142-3p promoted monocyte-derived DCs (moDCs) to secrete CCL2, CCL5, CXCL8, IL-6, TNF-α and other SLE-related cytokines. Moreover, overexpression of miR-142-3p in moDCs inhibited the proliferation of CD4^+^CD25^+^Foxp3^+^ Treg cells and recruited more CD4^+^ T cells, which impacted moDC-CD4+ T cell interactions (27). Regarding Tol-DCs, although a recent publication detailing that adoptive transfer of drug-induced Tol-DC1s and Tol-DC3s reported beneficial therapeutic effects in MRL-Fas^lpr^ lupus-prone mice (78), to date, there have been no relevant studies on the role of miRNAs in regulating DC tolerance in SLE.

### Rheumatoid Arthritis (RA)

RA is a chronic and inflammatory synovitis systemic autoimmune disease and is the most frequent autoimmune polyarthritis, with a lifetime prevalence of 3.6% in women and 1.7% in men (79, 80). Activation of DCs is involved in the pathogenesis of RA. Synovial fluid can contain both conventional CD1c+ and inflammatory CD1c+ cells, and these cells not only prime naive T cells (81) but also stimulate TLR7/8 ligands; in response, cytokines such as TNF are produced, thereby promoting synovial inflammation (82). Changes in the expression level of miRNAs can affect the abundance of DC surface receptors and thus regulate the maturation of DCs to change the inflammatory state in RA. A study found that CD1c+ DCs continuously expressed high levels of miR-34a, which inhibited the expression of cellular AXL, a tyrosine kinase receptor, thus contributing to the development of experimental arthritis. This expression of miR-34a may shift DCs towards a mature state, and mature DCs can support autoreactive T cells. Furthermore, in animal studies, compared with wild-type (WT) mice, miR-34a^−/−^ mice had a significantly lower incidence and severity of arthritis (28), which means that miR-34a inhibitors could be a potential treatment for RA. In addition, miRNAs can also affect helper T cell differentiation by regulating DCs, thus affecting the development of RA. Another study found that CD11C^+^av^+^ DCs induced Th17 cell differentiation. A possible mechanism has been proposed: decreased miR-363 expression in DCs from RA patients was shown to upregulate the expression of integrin av, which induced the activation of TGF-β and promoted the differentiation of Th17 cells (29); Th17 cells can exacerbate RA and are directly involved in cartilage and bone destruction (83).

### Sjögren’s Syndrome

Primary Sjogren’s syndrome (pSS) is an autoimmune disease characterized by inflammatory cells infiltrating multiple exocrine glands, such as salivary glands and lacrimal glands, and leads to a series of pathological manifestations, such as sicca keratoconjunctivitis and xerostomia (84). The number of pDCs in the peripheral blood of pSS patients is decreased (85), but in the target organ and salivary glands, the quantity of IFN-α-producing cells is increased (86, 87). Importantly, pDCs can also be activated by endogenous nucleic acids (88). Therefore, pDCs are considered to be the main contributor to the production of type I IFN in pSS and a key mediator of immunopathology. In addition, in pSS, multiple studies have shown that miRNAs are abnormally expressed in multiple tissues and cells of the human body, including purified immune cells, peripheral blood mononuclear cells (PBMCs) and salivary gland tissues (89, 90). In recent years, researchers have also noted the regulatory effects of miRNAs on DCs in pSS. Hillen et al. focused on 20 miRNAs that were differentially expressed between pDCs from patients with pSS and normal controls by an OpenArray quantitative PCR-based technique. In this study, abnormal regulation of the miRNome affected the type I IFN secretion and death of pDC from patients with pSS, and downregulation of pro-apoptotic factors such as miR-29a and miR-29c strengthened the survival of pDCs (30). Not only pDCs but also cDCs are involved in the pathological processes of pSS. cDC2s, which characteristically express CD1c, are the predominant cDCs in human blood, tissues, and lymphatic organs (8). Importantly, CD4+ T cells, the main target cells of cDC2s, play a crucial role in pSS immunopathology (91, 92). Ana P. Lopes et al. found that miR-708 and miR-130a expression in pSS cDC2s was downregulated after activation of some TLRs (TLR3 and TLR7/8), and this altered expression was involved in the pathogenesis of pSS. In addition, the secretion of inflammatory cytokines was increased. These results suggest that decreased expression of miR-130a and miR-708 can reflect cDC2 activation (31). Furthermore, miR-130a regulates the expression of MSK1, a targeted signalling protein overexpressed in cDC2s in pSS and an upstream mediator of NF-κB that regulates the secretion of some pro-inflammatory cytokines by cDC2s (31, 93).

### Inflammatory Bowel Disease (IBD)

A large number of microorganisms accumulate in the intestinal mucosa shortly after birth (94). Studies have shown that in the process of innate immune activation, specific miRNAs are upregulated, thereby affecting the innate response to microbial and viral infections (95). Mature DCs become highly specialized APCs when they encounter microbial products and inflammatory stimulation. Previous research has shown that lamina propria DCs may be associated with specific immune functions in the lamina propria and Peyer plaques (96). Therefore, miRNA-based regulation of DCs in intestinal immunity has gradually become a research focus. In one study, owing to the effects of enteric microorganisms, the expression of the miR-10a precursor was inhibited, which caused decreased expression of IL-12/IL-23p40 in DCs. In line with this finding, a miR-10a inhibitor promoted the expression of IL-12/IL-23p40. The gene encoding IL-12/IL-23p40, IL-12B, has been closely related to susceptibility to Crohn’s disease (CD) and somewhat related to susceptibility to ulcerative colitis (UC) (97–99). Another study determined whether abnormal expression of miR-10a in human DCs could inhibit the expression of NOD2, which is a prototypical member of the IL-12/IL-23P40 and nod-like receptor family. Furthermore, NOD2 can be activated by muramyl dipeptide (MDP) from bacteria (32, 100). Researchers have long believed that the NOD2 polymorphism is related to susceptibility to CD (101). Therefore, the regulation of DCs by miR-10a may also be one of the pathological mechanisms underlying IBD.

### Multiple Sclerosis

Multiple sclerosis is an autoimmune disease characterized by inflammatory demyelination of white matter in the central nervous system (CNS). The most commonly involved areas are the alba around the ventricle, optic nerve, spinal cord, brainstem and cerebellum. Through analyses of experimental autoimmune encephalomyelitis (EAE) and multiple sclerosis (MS) mouse models, researchers have found that MoDCs, which are Ly6c^hi^CD11b^+^CD11c^+^, are important CNS-infiltrating cells (102, 103). Another publication reported that miR-223, which is among the upregulated miRNAs in MS patients (104), plays an important role in inflammation in the CNS by controlling the level of MoDC-secreted Th17-polarizing cytokines (including IL-1β, IL-6 and IL-23) to regulate the induction of the Th17 response (33). Hoye et al. focused on the elevated expression of miR-31 in DCs that migrate through the blood-brain barrier *in vitro*. These results suggest that miR‐31 may have potential regulatory effects on DC migration in the CNS during EAE (34). In addition, a recent publication found that miPEP155 can regulate the antigen-presenting capacity of dendritic cells in an inflammatory environment and has a good therapeutic effect on two autoimmune diseases in mouse models of psoriasis and multiple sclerosis (72).

### Systemic Sclerosis (SSc)

Systemic sclerosis (SSc) is an autoimmune disease characterized by fibrosis, vascular lesions, and immune dysfunction. pDCs infiltrate the skin of SSc patients and become chronically activated, leading to the secretion of IFN-α and CXCL4, which is characteristic of the disease (105). One publication noted that overexpression of miR-618 reduced the development of pDCs *in vitro* and enhanced the ability of cells to secrete IFN-α, suggesting that miR-618 may be an important epigenetic target for regulating immune system homeostasis in diseases characterized by a type I IFN signature (35).

### Autoimmune Myocarditis

As the main cause of sudden death and dilated cardiomyopathy in children and young adults, autoimmune myocarditis features aseptic inflammation of cardiac tissues, and miRNAs play a regulatory role in its induction by inducing the generation of Tol-DCs. A large number of animal models have proven that Tol-DCs can inhibit the occurrence and/or progression of autoimmune diseases through adoptive transfer of BMDCs into mouse models (106–108). A recent study found that the inflammation of heart tissue and poor heart function in experimental autoimmune myocarditis (EAM) mice were reversed after transfusion of miR-223-3p-overexpressing DCs, indicating that miR-223-3p is involved in inducing Tol-DCs and regulating tolerance in autoimmune myocarditis (36).

### Acute Graft-*Versus*-Host Disease (aGVHD)

Among immune tolerance-related diseases, acute graft-*versus*-host disease (aGVHD) is a major immune complication that occurs after allogeneic haematopoietic cell transplantation (allo-HCT) due to a series of cytokine storms initiated by the recipient (109). MiRNAs are small non-coding RNAs, and their role in regulating inflammation and innate and adaptive immune responses cannot be ignored. The expression of multiple target mRNAs can be regulated by the same miRNA (110). In recent years, some publications have focused on the crucial role of miRNA dysregulation in DCs in the GVHD pathomechanism. One study noted that miR-155 expression was increased in activated DCs, and the severity of GVHD in miR-155^−/−^ transplant recipients was decreased when DC migration and the level of inflammasome activation were attenuated (37). Stickel et al. revealed that miR-146a can negatively regulate the JAK-STAT signalling pathway in DCs, suggesting that miR-146a variants can significantly increase the risk of acute severe GVHD in human allo-HCT recipients (38). Another study identified a partial role of miR-29a in stimulating DCs through TLR7 and TLR8 (in mice and humans, respectively) to release pro-inflammatory cytokines TNF and IL-6, which are critical drivers of acute GVHD pathogenesis, and to increase T cell proliferation (39). These studies provide a new research paradigm for identifying more effective prevention and treatment strategies for acute GVHD.

## LncRNAs Regulate Dendritic Cell-Mediated Autoimmune and Immune Tolerance-Related Diseases

LncRNAs, with lengths over 200 nt, are a group of non-coding RNAs with structures similar to mRNAs but lack any significant open reading frames (111, 112). In addition, they play crucial roles in various biological processes, such as immune cell differentiation, apoptosis and immune responses (20, 113). Many lncRNAs can be induced by TLRs. For example, stimulation of TLR4 induces the expression of lincRNA-Cox2 in CD11C+ BMDCs (15). In the following sections, we summarize previous studies of lncRNAs affecting DCs in autoimmune diseases and transplantation immunity.

### Systemic Lupus Erythematosus (SLE)

LncRNAs may be involved in the molecular regulatory mechanisms in lupus (114). Li et al. focused on the expression of lnc-DC in SLE patients, which was significantly lower than that in healthy controls. In contrast, the lnc-DC level was higher in the lupus nephritis group than in the healthy control group. To identify the correlation between differentially expressed lncRNAs in MoDCs of SLE patients and the SLEDAI score, Wang et al. used lncRNA microarrays and qPCR and found that the expression levels of ENST00000604411.1 and ENST00000501122.2 were able to estimate the activity of SLE. Specifically, the expression of these two markers was positively correlated with the SLEDAI score (40). These results suggest that lnc-DC could be a new biomarker for SLE.

### Immune Tolerance

**Figure 2:**
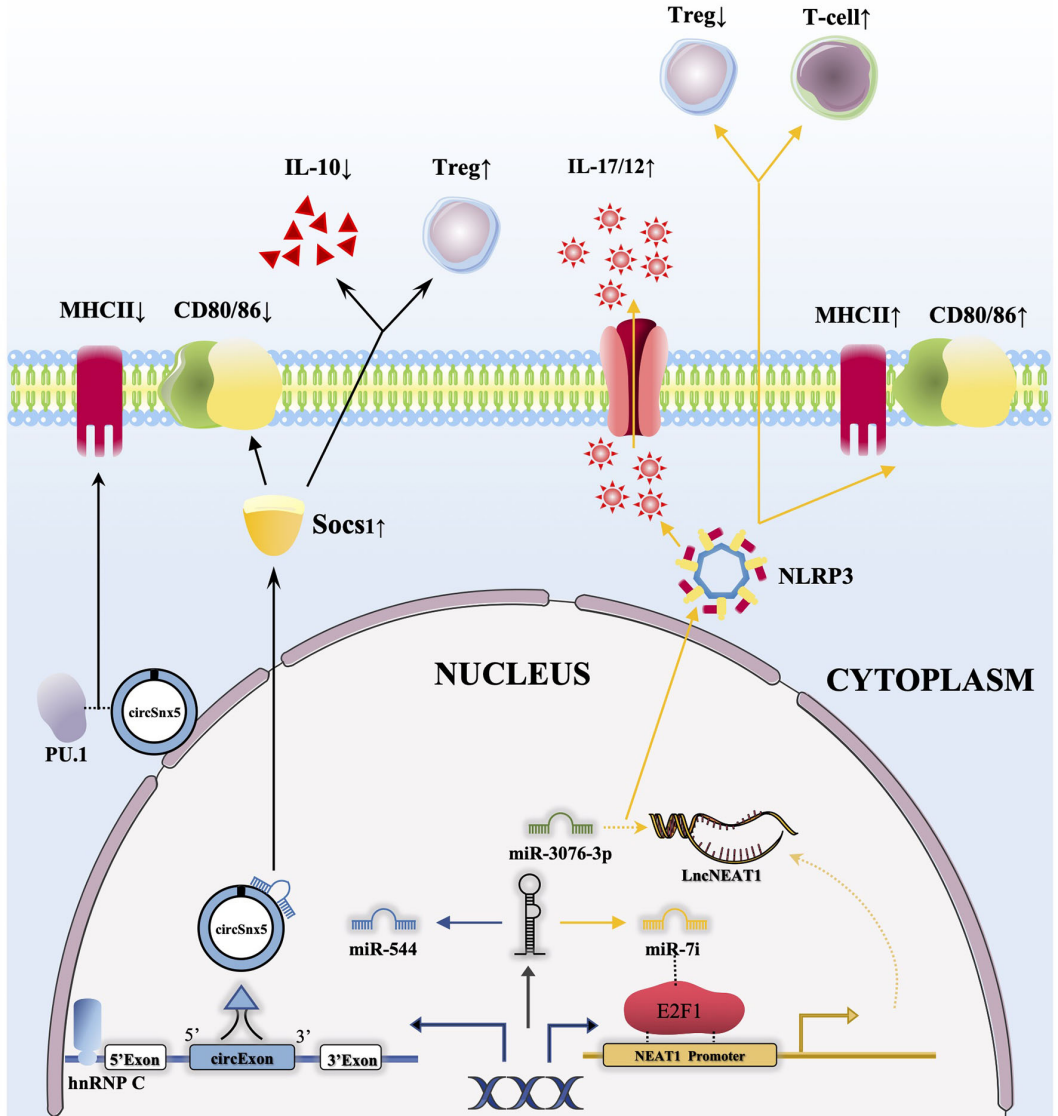
CircRNAs and lncRNAs regulate the function of DCs through the ceRNA network. 1) The combination of hnRNP C with circSnx5 promotes the expression of circSnx5 in DCs, and circSnx5 sponging with miR-544 reduces the inhibitory effect of miR-544 on Socs1, thus reducing the expression of CD80/86 and the secretion of IL-10 and increasing the number of Tregs. In addition, circSnx5 combined with PU.1 can directly reduce the expression of MHCII**;** 2) miRNA let-7i can regulate the expression of lncNEAT1 by binding E2F1, and lncNEAT1 is able to regulate NLRP3 inflammasome by inhibiting Mir-3076-3P, then increasing expression of MHCII/CD80/86, promoting secretion of IL-17/12 as well as reducing the number of Tregs and increasing the activation of T cells.

In transplantation immunity, abnormal lncRNA expression levels can affect the transformation of DCs into Tol-DCs. Yu et al. confirmed that the expression of the lncRNA NEAT1 was increased in mature DCs induced by LPS. As a ceRNA, NEAT1 regulated NLRP3 expression by affecting the activity of miR-3076-3P, and the expression of lncRNA NEAT1 could be regulated although E2F1 activity mediated by miR Let-7i **(** **Figure 2** **)**. Thus, transfusion of NEAT1-knockdown DCs into mouse models with EAM and heart transplantation reduced inflammatory cell infiltration, inhibited T cell proliferation, and increased the number of Treg cells (41). Another publication noted that the functional lncRNA MALAT1 is involved in Tol-DC induction and regulation of immune tolerance in heart transplantation and EAM. MALAT1 regulates the formation of Tol-DCs and immune tolerance by functioning as a miR155 sponge in the cytoplasm to promote DC-SIGN and IL10 production (42).

## Circular RNAs Regulate Dendritic Cell-Mediated Autoimmune and Immune Tolerance-Related Diseases

Circular RNAs are widely found in human and mouse genomes, so they are likely to be a common feature of eukaryotic gene expression and regulation, although they were previously ignored (115). In addition, they have been subsequently found in the genes of other animals, including flies and worms, by microarray analysis (116, 117). There is mounting evidence that circRNAs play an essential role in complex human pathologies. circRNAs have been used in some studies as new noninvasive biomarkers for certain autoimmune diseases (118). DCs are regarded as an important class of APCs in autoimmunity. DCs have been found to be involved in various autoimmune diseases and immune tolerance-related diseases; therefore, an in-depth study of the regulatory mechanisms by which circRNAs affect DCs will not only improve our understanding of the molecular mechanisms of these diseases but also make it possible to identify future treatments for them.

### Systemic Lupus Erythematosus (SLE)

Recent studies have suggested that circRNAs may play a regulatory role in SLE by serving as miRNA sponges (119, 120) and can be used as potential biomarkers for SLE (120). Another study confirmed that the circRNA hsa_circ_0045272 negatively regulates apoptosis and interleukin-2 secretion in SLE. There are other relevant studies on the regulation of DCs. For example, circHLA-C was shown to play a potentially important role in the pathogenesis of lupus nephritis by sponging miR-150. In addition, through GO analysis, it was found that upregulated circRNAs are involved in regulating the differentiation of DCs and other biological functions (43).

### Immune Tolerance

A large number of studies have shown that circRNAs play an important role in the immune system (121), and some circRNAs have been found to be abnormally expressed in DCs with different functions (21). The role of circRNAs in inducing Tol-DCs cannot be ignored. A recent publication found that circSnx5 could bind with miR-544 as a molecular sponge by analysing circSnX5-associated competing endogenous RNA (ceRNA) networks to weaken the inflammatory phenotype of DCs and enhance their tolerance in a heart transplantation mouse model (44) (**Figure 2**). In addition, some upstream regulatory factors may affect the expression of circRNAs to regulate the function of DCs. Another study studied growth differentiation factor 15 (GDF15)-induced Tol-DCs by inhibiting the circ_Malat-1 and NFκB signalling pathways (21). This study indirectly confirmed that the circRNA Malat-1 has a regulatory effect on DCs in immune tolerance.

## The Therapeutic Potential of Noncoding RNAs in Autoimmune Diseases

Changing the expression level of non-coding RNAs can further affect the process of autoimmune diseases through the regulation of DC function. As described above, the inflammatory response in SLE can be reduced by reducing the expression of miR-142-3p and miR-150 (27, 77). In addition, miR-29b and miR-29c can also enhance the effect of glucocorticoids on SLE by promoting pDC apoptosis (25). In addition, miR-142-3p, miR-363 and miR-29a change the proliferation level of Treg and T cells through regulation of DCs and then affect the level of inflammation in related autoimmune diseases (29, 39, 77). For the other two types of non-coding RNA (circRNA, lncRNA), representatively, CircSnx5 and lncNEAT1 can bind miRNA *via* a ceRNA network and change the inflammatory phenotypes of DCs in related autoimmune diseases (41, 44). In general, knockdown or overexpression of non-coding RNAs may be a novel potential therapeutic strategy for related autoimmune and tolerance-related diseases. In the development process of different autoimmune and tolerance-related diseases, it is of great potential to further understand the abnormal expression of non-coding RNAs and the regulation of these diseases through DCs, which can bring new therapeutic targets or strategies for these complex ones.

## Conclusion and Future Perspectives

Dendritic cells (DCs), typical APCs in the human body, play an important role in connecting innate immunity and adaptive immunity and affect the pathological mechanism of various immune diseases. Our understanding of non-coding RNAs has changed, and now, instead of being considered “junk” transcription products, they are recognized as functional regulators that mediate various cellular processes. This review highlights the regulatory effects and potential therapeutic targets targeted by DCs of abnormally expressed non-coding RNAs (miRNAs, lncRNAs, circRNAs) in autoimmune diseases and immune tolerance diseases. Although non-coding RNAs have been proven to be potential diagnostic and prognostic biomarkers, the specificity and sensitivity of most existing noncoding RNA biomarkers are still insufficient for clinical application. Further large-scale prospective clinical trials will validate and promote the clinical application of noncoding RNA biomarker candidates. Furthermore, the number and profundity of studies on the effects of lncRNAs and circRNAs on DCs in these diseases remain scarce. Despite these defects, further research on the regulatory mechanisms of non-coding RNA in target cells in specific diseases may provide a more solid foundation for diagnostic and therapeutic research in autoimmune diseases and immune tolerance diseases.

## Author Contributions

YL wrote the original draft, table and figure preparation. XW contributed to the conception and design of the study. FY and YZ: literature query. TY: editing. LY: supervision, review and editing. All authors contributed to the article and approved the submitted version.

## Funding

This work was supported by grants from the National Natural Science Foundation of China (No. 81770568 to LY).

## Conflict of Interest

The authors declare that the research was conducted in the absence of any commercial or financial relationships that could be construed as a potential conflict of interest.

## Publisher’s Note

All claims expressed in this article are solely those of the authors and do not necessarily represent those of their affiliated organizations, or those of the publisher, the editors and the reviewers. Any product that may be evaluated in this article, or claim that may be made by its manufacturer, is not guaranteed or endorsed by the publisher.

## Abbreviations

DCs, dendritic cells; pDCs, plasmacytoid dendritic cells; cDCs, myeloid/conventional dendritic cells; APCs, antigen-presenting cells; MPS, mononuclear phagocyte system; IRF, interferon regulatory factor; Treg, T regulatory cells; STAT3, Signal Transducers and Activators of Transcription 3; GDF15, growth differentiation factor 15; PRRs, pattern-recognition receptors; ConA, Concanavalin A; BDCA, blood dendritic cell antigen; TNF-α, tumour necrosis factor alpha; AIH, autoimmune hepatitis; Tol-DCs, Tolerogenic dendritic cells; MHC, major histocompatibility complex; Mcl-1, myeloid cell leukaemia-1; Bcl-2, B-cell lymphoma-2; TREM-1, Triggering receptor expressed on myeloid cells-1; TGF-β, transforming growth factor beta; MSK1, mitogen-and stress-activated protein kinase 1; NOD2, nucleotide-binding oligomerization domain 2; IBD, Inflammatory bowel disease; CD, Crohn’s disease; UC, ulcerative colitis; MDP, muramyl dipeptide; pSS, primary Sjogren’s syndrome; RA, Rheumatoid arthritis; SLE, Systemic lupus erythematosus; ATP, adenosine triphosphate; IDO, indoleamine 2,3-dioxygenase; CNS, central nervous system; EAE, experimental autoimmune encephalomyelitis; MS, multiple sclerosis; MoDCs, monocyte derived dendritic cells; SSc, Systemic sclerosis; EAM, experimental autoimmune myocarditis; aGVHD, acute graft-versus-host disease; allo-HCT, allogeneic haematopoietic cell transplantation; LPS, Lipopolysaccharide; E2F1, E2F transcription factor 1; ceRNA, competing endogenous RNAs.
